# ProsTAV, a clinically useful test in prostate cancer: an extension study

**DOI:** 10.1007/s00345-024-05098-8

**Published:** 2024-07-10

**Authors:** Enrique Gómez-Gómez, Juan Ignacio Martínez-Salamanca, Fernando Bianco, Brian J Miles, Javier Burgos, Juan Justo Quintas, Roque Cano-Castiñeira, Álvaro Gómez-Ferrer, Alfredo Rodríguez-Antolín, Gilberto Chéchile, Luis Fernández, Almudena Martín, Paloma Hidalgo, Mónica Parramón

**Affiliations:** 1 Department of Urology, Hospital Universitario Reina Sofía, Universidad de Córdoba, IMIBIC, Córdoba, Spain; 2 LYX Urology Institute, Madrid, Spain; 3 Urological Research Network, Miami Lakes, FL USA; 4 Urologic Oncology, Houston Methodist Hospital, Houston, TX USA; 5 Department of Urology, Hospital Universitario Ramón y Cajal, Universidad de Alcalá, Instituto Ramón y Cajal de Investigación Sanitaria, Madrid, Spain; 6 ROC Clinic y HM Hospitales, Madrid, Spain; 7 Department of Urology, Hospital Infanta Margarita, Córdoba, Spain; 8 IVO Instituto Valenciano de Oncología (IVO), Valencia, Spain; 9 Department of Urology, Hospital Universitario Doce de Octubre, Madrid, Spain; 10 Instituto Médico Tecnológico, Barcelona, Spain; 11 Life Length, S.L., Madrid, Spain

**Keywords:** Prostate cancer, Biomarker test, Telomere, Predictive model, Biopsy

## Abstract

**Purpose:**

To assess the clinical performance of ProsTAV^®^, a blood-based test based on telomere associate variables (TAV) measurement, to support biopsy decision-making when diagnosing suspicious prostate cancer (PCa).

**Methods:**

Preliminary data of a prospective observational pragmatic study of patients with prostate-specific antigen (PSA) levels 3–10 ng/ml and suspicious PCa. Results were combined with other clinical data, and all patients underwent prostate biopsies according to each center’s routine clinical practice, while magnetic resonance imaging (MRI) before the prostate biopsy was optional. Sensitivity, specificity, positive and negative predicted values, and subjects where biopsies could have been avoided using ProsTAV were determined.

**Results:**

The mean age of the participants (*n* = 251) was 67.4 years, with a mean PSA of 5.90 ng/ml, a mean free PSA of 18.9%, and a PSA density of 0.14 ng/ml. Digital rectal examination was abnormal in 21.1% of the subjects, and according to biopsy, the prevalence of significant PCa was 47.8%. The area under the ROC curve of ProsTAV was 0.7, with a sensitivity of 0.90 (95% CI, 0.85–0.95) and specificity of 0.27 (95% CI, 0.19–0.34). The positive and negative predictive values were 0.53 (95% CI, 0.46–0.60) and 0.74 (95% CI, 0.62–0.87), respectively. ProsTAV could have reduced the biopsies performed by 27% and showed some initial evidence of a putative benefit in the diagnosis pathway combined with MRI.

**Conclusions:**

ProsTAV increases the prediction capacity of significant PCa in patients with PSA between 3 and 10 ng/ml and could be considered a complementary tool to improve the patient diagnosis pathway.

## Introduction

Prostate cancer (PCa) is the most common neoplasia among men, except for skin cancer. An estimated 288,300 males in the US and 473,344 in Europe will receive a PCa diagnosis in 2023 [1]. Worldwide, an estimated 1.4 million people were diagnosed with PCa in 2020, making it the fourth most prevalent cancer. Since 2014, overall and advanced-stage PCa incidence rates have increased by around 3% and 5% per year, respectively [2].

Although prostate-specific antigen (PSA) is the most widely used biomarker for early diagnosis and follow-up [3], its specificity for PCa is poor, leading to unnecessary biopsies, over-diagnosis, and over-treatment [4]. European scientific societies have promoted using biomarker tests, mainly from blood, urine, imaging, or tissue-based [5]in patients with known PSA values to improve specificity and avoid over diagnosing non-significant PCa (ISUP 1) [6]. However, their clinical utility should be explored, including screening and pre-biopsy selection and comparison, or in combination with tools such as risk nomograms and magnetic resonance imaging (MRI) [7].

MRI is a valuable tool in the diagnosis of clinically significant prostate cancer (csPCa) identifying biopsy candidates [8]. However, a lack of resources in most radiology departments makes MRI difficult for many patients. In addition to the necessary training of MRI specialists, standardization of the technique is required [9] given the significant variability in scan quality and the experience and skill of the interpretation by the radiologist.

The European Commission has recommended developing prediction models of PCa based on multiple biomarkers. However, in most cases, current biomarkers lack the required validation to be implemented in routine clinical practice [10].

Specific telomere length measurements in peripheral blood leucocytes have been associated with higher PCa risk and a greater risk of aggressive disease [11]. Telomere Analysis Technology® (TAT®) combines advanced techniques to assess telomere-associated variables (TAV) in cells [12]. ProsTAV is a CE-marked in vitro diagnostic, minimally invasive, and easy-to-implement biomarker test developed by Life Length. It was developed using TAV as a complementary tool to diagnose csPCa for patients with elevated PSA (between 3 and 10 ng/ml).

A recent study showed that subjects with PSA levels between 3 and 10 ng/ml may benefit from using ProsTAV [13] demonstrating its potential to reduce the biopsies in subjects of uncertain risk by 33%. Here, we present the preliminary results of the validation extension study to determine the efficacy of ProsTAV in diagnosing csPCa.

## Methods

A multicenter prospective observational study was performed on patients suspicious of PCa who underwent prostate biopsy from June 2022 to March 2023 in ten hospitals in Spain and the US. Ethical approval was granted by local committees (clinicaltrials.gov NCT04124900), and informed consent was obtained from all participants.

### Population

Eligible patients were Caucasian males ≥ 18 years of age, at PCa-risk (PSA between 3 and 10 ng/ml), and biopsy candidates according to current clinical practice [5]. Patients treated with alpha-5-reductase inhibitors, with any active liver, lung, or kidney disease, severe infection, mental impairment to follow the study procedures, or other active neoplasia were excluded.

### Prostate-related assays

PSA and free PSA values and digital rectal examination (DRE) were performed according to local standard practice. All patients underwent a transrectal or trans perineal prostate biopsy, systematic or with template +/- target biopsy, in case of suspicious lesion on MRI. Uropathologists from each major referral center evaluated specimens following the recommendations of the International Society of Urological Pathology (ISUP) [14]. Patients with clinically significant PCa were those with ISUP > 1 (Gleason score ≥ 7). Each participating hospital followed local MRI protocols based on PIRADS (prostate imaging reporting and data system by the International Prostate MRI Working Group) scoring 2.1 [15].

### Telomere analysis technology- ProsTAV

TAT determines the telomeric length in the cell nucleus of isolated blood lymphocyte samples. It labels telomeres by using the *in-situ* hybridization technique (HT Q-FISH) with specific fluorescently labeled peptide nucleic acid (PNA) probes, detecting them on a high-throughput Perkin Elmer (Revvity) Phenix microscope [16]. Data were analyzed using proprietary software to generate all TAVs validated through the same CLIA and ISO15189 laboratory standards. The validation analyses were conducted on samples from the included subjects on mononuclear cells isolated from peripheral blood. These tests were performed in the laboratories of Life Length, S.L. (Madrid, Spain)

ProsTAV is a risk estimation test based on algorithm-derived models that use TAV as a biomarker diagnosing PCa (also called “risk score”) based on a refined model. Data derives from telomeric measurements and the patient’s clinical history to indicate whether the individual is at low risk and, therefore, avoid performing a prostate biopsy for the diagnoses of PCa. A biopsy is only recommended when the ProsTAV score is above 10% [13]. ProsTAV was developed by multivariate logistics regression based on clinical variables (PSA, free PSA, age, previous biopsy, and DRE) and TAV. The latter consists of three “short telomere” (percentage of telomeres of a sample with a length under a specific threshold) and three “short cell” (percentage of cells of a sample whose telomeres average a length under a specific threshold) variables [13]. The samples were handled according to ProsTAV’s technical specifications, so the cold supply chain was always maintained, and the lymphocytes were isolated within 72 h of sample extraction from the patient [17].

### Statistic methods

Descriptive statistics were used to present baseline characteristics of enrolled participants, including age, PSA, free PSA, PSAD, MRI PIRADS score, and ProsTAV results. Data are expressed as means and standard deviation unless otherwise stated. All tests were considered two-tailed; p-values below 0.05 were considered statistically significant. Statistical analysis of the data was performed using SPSS® software R.8.01 and R V.4.3.1 (R Foundation for Statistical Computing, Vienna, Austria; https://www.R). The predictive capacity and accuracy of ProsTAV were summarized by receiver operating characteristic curves (ROC) through another open-access calculator (https://datatab.es/statistics-calculator/roc-calculator).

## Results

A total of 251 patients were included in this preliminary analysis. Their characteristics and csPCa results according to the ISUP grading system are shown in Table 1.

**Table 1:** Characteristics of the study population

Variable	Total (*N* = 251)
Age, years, mean (SD)	67.4 (8.4)
PSA, ng/ml, mean (SD)	5.9 (1.6)
Free PSA, %, mean (SD)	18.9 (8.8)
PSA density, ng/ml, mean (SD)	0.14 (0.09)
Suspicious DRE, N (%)	21 (8.4)
csPCa, N (%)	120 (47.8)
Non-significant PCa	32 (17.2.)
No cancer	99 (39.4)
Cylinders affected by tumor, N, mean (SD)	4.6 (3.0)
% of Maximum affectation, N, mean (SD)	50.0 (30.3)
Tumor length of the most affected cylinder, mm, mean (SD)	50.0 (30.3)
ISUP 2 (*n* = 44, 17.5%), mm, mean (SD)	7.04 (5.63)
ISUP 3 (*n* = 43, 17.1%) mm, mean (SD)	7.18 (5.29)
ISUP 4 (*n* = 21, 8.4%), mm, mean (SD)	9.58 (4.82)
ISUP 5 (*n* = 12, 4.8%), mm, mean (SD)	13.00 (7.06)
**mp MRI**	*N* = 207
PIRADS, N (%)	
1	14 (6.8)
2	21 (10.1)
3	52(25.1)
4	94 (45.4)
5	26 (12.6)

Abbreviations: csPCa, clinically significant prostate cancer; DRE, digital rectal examination; IQR, interquartile range; ISUP, International Society of Urological Pathology; mp MRI, multiparametric magnetic resonance imaging; PCa, prostate cancer; PIRADS, prostate imaging reporting and data system; PSA, prostate-specific antigen; SD, standard deviation.

### ProsTAV performance and clinical validation

**Fig. 1:**
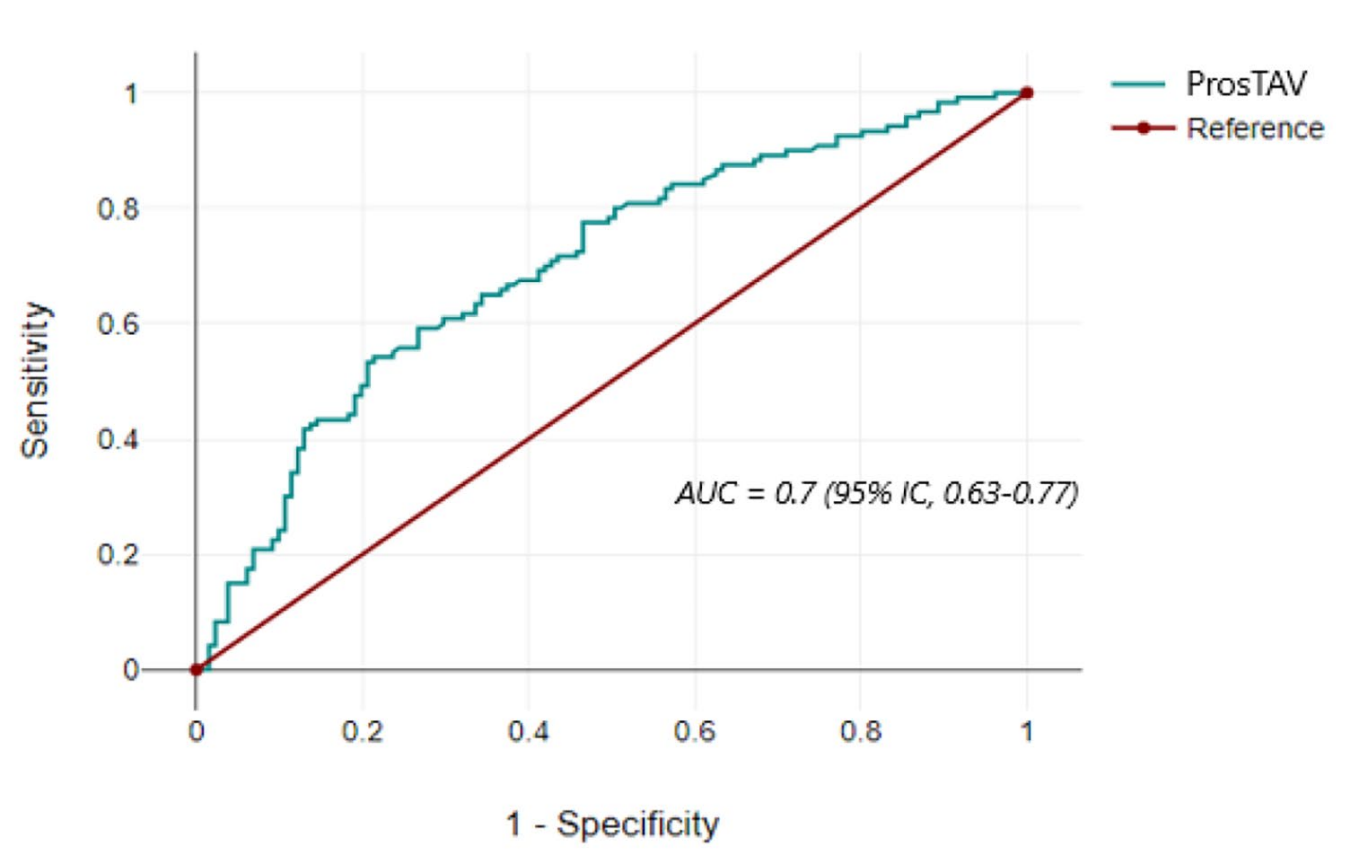
ROC curve analysis shows ProsTAV effectiveness in the real-world cohort. Abbreviations: AUC, area under the curve; CI, confidence interval

The two main values analyzed for the precision and consistency of ProsTAV, i.e., sensitivity and specificity, were 0.90 and 0.27, respectively. The positive predictive value (PPV) was 0.53, while the negative predictive value (NPV) was 0.74. When data was analyzed in the subgroup of age > 50 years and PSA > 4 ng/ml (*n* = 226), the sensitivity was 0.92, the specificity 0.26, NPV 77%, and false negative rate (FNR) 8%. No aggressive cancers (Gleason score ≥ 8) were missed with ProsTAV. The predictive capacity and accuracy of ProsTAV are summarized in the ROC curve (AUC = 0.7; 95% IC; 0.63–0.77) (Fig. 1).

### ProsTAV likelihood ratio results

The ProsTAV positive and the negative likelihood ratios found were 1.23 and 0.37, respectively. In our sample, in those subjects with a positive ProsTAV result (> 10), the probability of csPCa was 53%. Conversely, if the patient showed a negative ProsTAV result (≤ 10), the probability of having csPCa was 25%, gaining an additional 23% of the likelihood for detection.

### ProsTAV® findings versus biopsy

When comparing the results obtained by the biomarker test versus those provided by the biopsy, it was observed that ProsTAV was accurate in 143 patients (57%), 108 patients with csPCa, and 35 healthy individuals. In contrast, ProsTAV did not match the biopsy results in 12 patients with csPCa and 96 healthy individuals. Thus, ProsTAV failed to identify 10% of the csPCa (sensitivity). On the other hand, since there were 131 individuals who, according to the biopsy, did not present csPCa, and ProsTAV identified 35 of them, the biomarker test could have avoided 27% of the biopsies performed.

### ProsTAV findings versus MRI

**Fig. 2:**
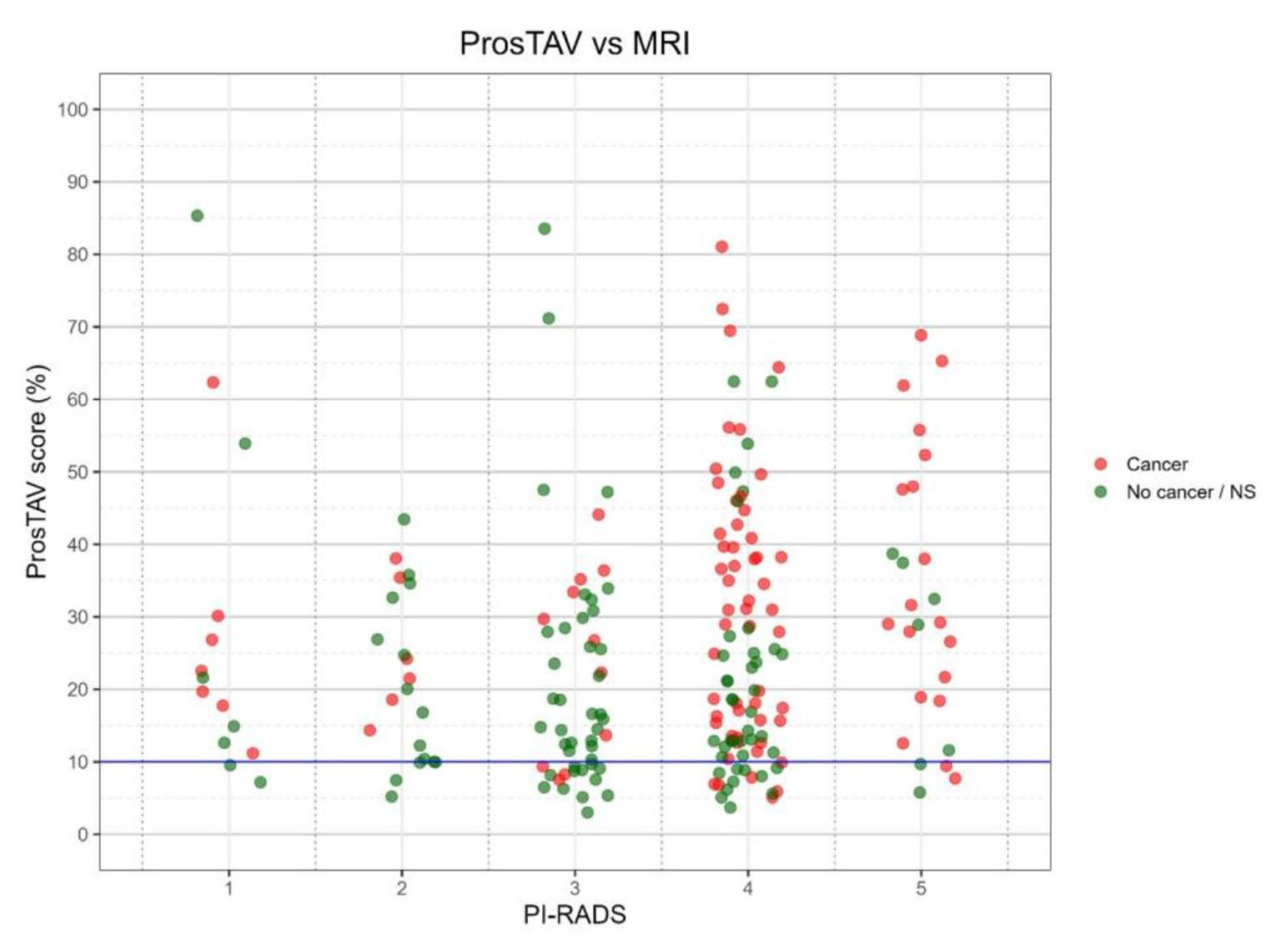
Comparison of the ProsTAV result and the PI-RADS score obtained by MRI. Patients were classified as having csPCa or non-significant PCa after biopsy. The ProsTAV score threshold is indicated as a solid line. Abbreviations: MRI, magnetic resonance imaging; PI-RADS, prostate imaging reporting, and data system; NS, non-significant PCa

MRI was performed on 207 (82.5%) subjects (Table 1). This technique showed that 24 patients were not imaging suspicious for cancer. The positive ProsTAV result (score > 10) would have rescued 21 patients in the cohort who had negative or equivocal MRI (PIRADS ≤ 3), and biopsy proved csPCa (Fig. 2). Besides, the prediction of combining ProsTAV score and MRI in the subgroup of patients with PI-RADS ≥ 3 results in an AUC equal to 0.74 (95% CI; 0.664–0.813).

The detection rate between biopsy, imaging, and ProsTAV results for those who would have been potentially selected for biopsy and those who would not are detailed in Fig. 2. According to these, MRI would have lost seven, six, and eleven (*n* = 24) patients with PIRADS 1, 2, and 3, respectively, from which ProsTAV would have correctly sent to biopsy seven, six, and eight (*n* = 21), respectively.

## Discussion

This study offers external validity results for the ProsTAV biomarker test, proving that ProsTAV could have reduced the biopsies performed significantly and showed some benefit in complementing MRI in the diagnosis pathway. Our investigation confirms the clinical utility of ProsTAV in managing patients at risk for csPCa in routine clinical practice. In our investigation, 47.8% of patients had csPCa, a higher number than in previous studies of ProsTAV (19.1% in the ONCOCHECK study [12] and 23.9% in the previous algorithm validation study [13]. This is very likely since selected patients will be planned for biopsy. In fact, among MRI results, PIRADS 4 was the most frequent. The population was well distributed in different PCa stages, so adequate comparative results can be drawn. The study population was elderly, with similar suspicious DRE results to those found in the former study [13].

The sensitivity, specificity, PPV, and NPV results obtained by ProsTAV in our cohort align with those previously published [13] (identical sensitivity, specificity 0.27 and 0.33, PPV 0.53 and 0.29 and NPV 0.74 and 0.91, respectively), validating the algorithm in a clinical pragmatic cohort. In addition, the AUC provided by the ROC curve of this study is consistent with the previous study (0.7 both). Differences in the PPV and NPV data can be explained by the different PCa prevalences. Other diagnostic tests are also affected by prevalence, as observed in biomarkers MDX [18], PHI [19] PCA3 [20], or even MRI [21].

It should be noted that the clinical validation data obtained for the subgroup of men > 50 years and PSA > 4 ng/dL are better than those obtained in the overall cohort. Specifically, the analytical validity data regarding sensitivity, NPV, and FNR are slightly improved. This is relevant because this subpopulation is tested on similar biomarkers [22, 23]. It is interesting to contextualize it versus the analytical values established by other biomarkers tests marketed [24–27]. ProsTAV identified all cases of aggressive PCa in the population studied, and the missed cases, according to ProsTAV, in subjects with Gleason 7, were higher in those with the best prognosis, i.e., Gleason 7 (3 + 4). All subjects with ISUP4 and ISUP5 values showed a positive ProsTAV score (> 10). Similarly, the biomarker test accurately diagnosed csPCa in 96.4% of the individuals with ISUP2 and 98.8% of those with ISUP3.

Likelihood ratios enhance the evaluation of the clinical value of test results by integrating all the raw data [28, 29]. Our cohort established 47.8% as the odds of having csPCa, by biopsy, the gold standard technique. After using ProsTAV, this probability is adjusted. Thus, in those patients with a positive ProsTAV result, the probability of having csPCa is 53% (5% higher), indicating moderate to high efficiency of the test [30], so it can be established that a positive ProsTAV is 5.2% more likely to have csPCa than a person who has not been tested. On the other hand, a negative ProsTAV result indicates a 25% probability of csPCa. Thus, a negative ProsTAV result is 27.2% less likely to involve csPCa than a person not tested, indicating ProsTAV is an efficient test [30].

Despite the current focus on MRI, it needs to be more precise to replace biopsy, mainly because of its variable accuracy [21], so ProsTAV could contribute to decision-making. In fact, 13 patients (6.28%) with csPCa showed PIRADS 1 or 2, while the biomarker test could identify accurately through a ProsTAV score > 10.

Whether patients with a PIRADS 3 lesion require a biopsy remains controversial, PIRADS 4 or 5 lesions do so [31, 32]. These negative MRI-selected patients likely had high-risk usual variables such as elevated PSA or PSA density, a low free PSA, or a strong family history. It should be considered that ProsTAV also considers some of these biomarkers/factors. Also, in this group, ProsTAV could be a complementary tool since 24 patients (11.59%) would not have been sent to biopsy according to their PIRADS results 3 (*n* = 12, 5.80%) or 4 and 5 (*n* = 12, 5.80%). In addition, the prediction of combining ProsTAV and MRI data in patients with suspicious lesions (PI-RADS ≥ 3) results in a higher AUC value increasing the diagnostic ability (0.74 vs. 0.70).

Awaiting results in clinical practice, this preliminary analysis supports the clinical utility of ProsTAV as a biomarker test. Our cohort, with unconfirmed diagnostic suspicion, has had in ProsTAV a tool to help identify biopsy needs. This would avoid causing patients to undergo this procedure unnecessarily and save healthcare resources and costs.

There might be a selection bias of patients caused by the pragmatic cohort of already selected patients for prostate biopsy. In our study, subjects were considered at risk for PCa if PSA > 3 ng/ml and/or positive DRE. However, PCa could also be suspected with PSA values < 3 ng/ml in some countries and have not been included in the present cohort. Therefore, a lower limit of PSA needs to be studied in the future, and the biomarker’s putative role in a not previously selected PIRADS 1–3 population. Besides, variability in the biopsy methodology could be an issue, although positive from a pragmatic point of view. There is a sparse number of patients with prostatectomy specimens to be evaluated, but another study, specifically in patients with prostatectomy, to evaluate the biomarker from a prognostic perspective is planned.

To conclude, and within the scope of the clinical utility of ProsTAV as a biomarker test for PCa, the current research shows the validation of the data obtained in the study used to develop the algorithm. ProsTAV could contribute to MRI in the current diagnoses’ pathway of PCa, and this possibility needs to be further explored in future ampler prospective trials.
